# Адаптация опросника 12-item Medication Adherence Scale (шкала приверженности лечению) на русскоязычной выборке пациентов с сахарным диабетом 1 и 2 типа

**DOI:** 10.14341/probl13372

**Published:** 2024-09-15

**Authors:** В. Е. Епишин, М. Ф. Калашникова, Н. В. Лиходей, И. Б. Бондарева, А. М. Каурова, М. В. Тулупова, Н. А. Николаев, В. В. Фадеев

**Affiliations:** Первый МГМУ имени И.М. Сеченова (Сеченовский Университет); Первый МГМУ имени И.М. Сеченова (Сеченовский Университет); Первый МГМУ имени И.М. Сеченова (Сеченовский Университет); Российский университет дружбы народов им. Патриса Лумумбы; Первый МГМУ имени И.М. Сеченова (Сеченовский Университет); Первый МГМУ имени И.М. Сеченова (Сеченовский Университет); Омский государственный медицинский университет; Первый МГМУ имени И.М. Сеченова (Сеченовский Университет)

**Keywords:** приверженность лечению, 12-item Medication Adherence Scale (MAS-12), сахарный диабет 1 типа, сахарный диабет 2 типа, Российский опросник количественной оценки приверженности к лечению (КОП-25), Опросник оценки приверженности медикаментозному лечению

## Abstract

**ОБОСНОВАНИЕ:**

ОБОСНОВАНИЕ. Низкая приверженность лечению у пациентов с сахарным диабетом 1 типа (СД1) и сахарным диабетом 2 типа (СД2) препятствует эффективному применению антидиабетических средств и достижению оптимального гликемического контроля, снижая качество их жизни и исходы. Оценка приверженности лечению с использованием опросника может помочь выявить и устранить факторы и барьеры, негативно влияющие на соблюдение врачебных рекомендаций и удовлетворенность лечением.

**ЦЕЛЬ:**

ЦЕЛЬ. Провести языковую и культурную адаптацию опросника 12-item Medication Adherence Scale (MAS-12) и оценить психометрические свойства русскоязычной версии опросника MAS-12 среди пациентов, страдающих СД1 и СД2.

**МАТЕРИАЛЫ И МЕТОДЫ:**

МАТЕРИАЛЫ И МЕТОДЫ. Проведено обследование 198 пациентов с СД1 и СД2, включающее самостоятельное заполнение опросника MAS-12 на русском языке. Средний возраст: 47,1±18,62 года, доля женщин — 76%. Средняя продолжительность заболевания: 13,08±10,05 года. Оценка конструктной валидности анкеты MAS-12 проводилась методом конфирматорного факторного анализа. В качестве внешнего критерия для оценки конвергентной валидности использовалась методика КОП-25 — Российский опросник для количественной оценки приверженности лечению (КОП-25). Надежность опросника MAS-12 оценивалась с помощью коэффициента внутренней согласованности Кронбаха и повторного тестирования участников спустя 1–4 месяца.

**РЕЗУЛЬТАТЫ:**

РЕЗУЛЬТАТЫ. Факторная структура опросника MAS-12 впервые воспроизводится на российской выборке пациентов с СД. Рекомендуемые показатели пригодности измерительной модели (CFI=0,983, RMSEA=0,049, TLI=0,968) достигаются при исключении двух вопросов (9 и 12), не продемонстрировавших статистически достоверного вклада в соответствующие им субшкалы. Внутренняя согласованность субшкал (α ϵ [0,522; 0,857]) и опросника в целом (α=0,766) была оценена как достаточная. Получены значимые корреляции адаптируемой методики и ее субшкал со шкалами опросника КОП-25. Наиболее тесные связи (r ϵ [0,333; 0,431], p<0,010) наблюдаются со шкалами КОП-25, относящимися к лекарственной терапии, что говорит о хорошей внешней валидности адаптируемой методики.

**ЗАКЛЮЧЕНИЕ:**

ЗАКЛЮЧЕНИЕ. Русскоязычная версия анкеты MAS-12 «Опросник оценки приверженности медикаментозному лечению» (ПМЛ-10), состоящая из 10 вопросов, обладает хорошими психометрическими свойствами, является валидным и надежным инструментом для оценки приверженности лечению среди больных с СД1 и СД2 и может быть рекомендована к применению в клинической практике, в том числе для мониторинга приверженности лечению на территории России.

## Abstract (en)

**BACKGROUND:**

BACKGROUND: Poor adherence to treatment among patients with type 1 diabetes mellitus (T1DM) and type 2 diabetes mellitus (T2DM) hinders the effective use of antidiabetic agents and the achievement of optimal glycemic control, reducing their quality of life and outcomes. Assessing adherence to treatment using a questionnaire can help identify and eliminate factors and barriers that negatively affect adherence to medical recommendations and satisfaction with treatment.

**AIM:**

AIM: To conduct linguistic and cultural adaptation of the 12-item Medication Adherence Scale (MAS-12) questionnaire and evaluate the psychometric properties of the Russian version of the MAS-12 questionnaire among patients suffering from T1DM and T2DM.

**MATERIALS AND METHODS:**

MATERIALS AND METHODS: A survey of 198 patients with T1DM and T2DM was carried out, including self-completion of the MAS-12 questionnaire in Russian. Average age: 47.1±18.62 years, proportion of women — 76%. Average duration of the disease: 13.08±10.05 years. The construct validity of the MAS-12 questionnaire was assessed using confirmatory factor analysis. As an external criterion for assessing convergent validity, the KOP-25 method was used - the Russian Questionnaire for Quantitative Assessment of Treatment Adherence (KOP-25). Reliability of the MAS-12 was assessed using Cronbach’s α internal consistency and participant retest after 1 to 4 months.

**RESULTS:**

RESULTS: The factor structure of the MAS-12 questionnaire is reproduced for the first time on a Russian sample of patients with diabetes. Recommended fit indicators for the measurement model (CFI=0.983, RMSEA=0.049, TLI=0.968) were achieved by excluding two items (9 and 12) that did not demonstrate statistically significant contributions to their respective subscales. The internal consistency of the subscales (α ϵ [0.522; 0.857]) and the questionnaire as a whole (α=0.766) was assessed as sufficient. Significant correlations of the adapted methodology and its subscales with the scales of the KOP-25 questionnaire were obtained. The closest connections (r ϵ [0.333; 0.431], p<0.010) are observed with the COP-25 scales related to drug therapy, which indicates good external validity of the adapted methodology.

**CONCLUSION:**

CONCLUSION: The Russian version of the MAS-12 questionnaire “Questionnaire for assessing adherence to medication treatment” (PML-10), consisting of 10 questions, has good psychometric properties, is a valid and reliable tool for assessing medication adherence among patients with T1DM and T2DM and can be recommended for use in clinical practice, including for monitoring treatment adherence in Russia.

## ОБОСНОВАНИЕ

Приверженность лечению — понятие, отражающее степень соблюдения пациентом предписанных ему инструкций и назначенной схемы лечения [1]. Оно включает в себя не только то, насколько точно человек следит за дозой препарата в каждый прием, но также учитывает явления отказа или временного прекращения приема препарата, случаи несоблюдения предписанной диеты, режима и другие параметры [2][3].

Проблема приверженности лечению существует в медицине с самых ее истоков, и в настоящее время, в эпоху активного развития фармакотерапии, ее значение существенно возросло [4]. Соблюдение лечебных рекомендаций — важнейшая составляющая практически любого терапевтического плана, во многом определяющая результаты терапии [5]. Именно низкая приверженность лечению является одним из ведущих факторов, обуславливающих недостаточную эффективность терапии и недостижение терапевтических целей, снижение качества жизни пациентов, высокие риски развития осложнений, госпитализации и смертности [6][7][8]. Особенно актуальна данная проблема в случае хронических заболеваний, поскольку в этом случае человеку необходимо принимать препараты и соблюдать схему лечения долгие годы, а зачастую и всю жизнь [9]. Кроме того, показатели приверженности необходимо учитывать при разработке новых лекарственных препаратов.

ВОЗ выделяет пять групп факторов или «барьеров», которые, взаимно влияя друг на друга, формируют степень приверженности каждого конкретного человека: психологические — связанные с личностью пациента; клинические — связанные непосредственно с заболеванием и его особенностями; факторы, связанные с врачом и системой здравоохранения в целом; факторы, связанные с особенностями терапии; совокупность социально-экономических факторов [4]. Данные факторы важно учитывать при разработке той или иной методики, направленной на оценку приверженности при различных нозологиях.

Существуют прямые и косвенные методы изучения приверженности лечению. К прямым обычно относят различные анализы жидкостей организма (крови, мочи), однако подобные способы не слишком показательны при пожизненный терапии, не обнаруживают некоторые лекарственные препараты и, кроме того, не всегда могут быть этичны и зачастую дорого стоят. Вследствие этого большая часть существующих данных о приверженности лечению основана на сборе косвенных и субъективных признаков: дневниках самоотчета, датах выдачи рецептов и количестве таблеток, интервью и т.д. [5].

Наиболее удобным способом оценки приверженности остаются валидизированные диагностические шкалы — опросники и анкеты [11]. На данный момент существует достаточно большое количество опросников, оценивающих приверженность лечению. Одним из наиболее известных и распространенных диагностических шкал является тест Мориски-Грин. Изначально данная методика состояла всего из 4 вопросов, но в дальнейшем авторы расширили ее до 8 пунктов [12]. Главное преимущества теста Мориски-Грин — это простота как восприятия, так и обработки, однако результаты ряда исследований указывают, что психометрические показатели усовершенствованной версии все еще остаются спорными (чувствительность — 51%, отрицательная прогностическая способность — 43%) [13].

Значительно более высокой чувствительностью обладает Краткий лекарственный опросник (BMQ — Brief Medication Questionnaire) [14]. Он весьма лаконичен, удобен для использования в клинической практике и направлен не только на измерение приверженности к приему лекарственных препаратов, но и на оценку уверенности пациента в необходимости проведения терапии. Несмотря на это, данный опросник остается не слишком популярным среди исследователей.

Опросник MARS (Medication Adherence Report Scale) был разработан в двух версиях: из 5 и из 10 пунктов [15]. Методика лаконична и удобна в использовании, а кроме того, может применяться при оценке приверженности у людей с психическими расстройствами. Благодаря перечисленным преимуществам опросник стал весьма популярным и был переведен на несколько языков, однако практика показывает, что MARS также имеет спорные показатели валидности при применении его на людях с различными хроническими заболеваниями [16].

Шкала «самоэффективности» в отношении применения лекарств SEAMS (The Self-Efficacy for Appropriate Medication Use Scale) — методика, показывающая хорошие результаты надежности при оценке приверженности у людей с различными хроническими заболеваниями, важным преимуществом которой, помимо прочего, является возможность ее применения в случае низкой грамотности пациентов [17]. Однако, несмотря на видимые достоинства, опросник SEAMS требует большего времени на заполнение, чем рассмотренные ранее опросники, и обладает относительно сложной системой подсчета результатов, что затрудняет его широкое применение в реальной клинической практике [15].

Российский опросник для количественной оценки приверженности лечению (КОП-25), разработанный Н.А. Николаевым и Ю.П. Скирденко, демонстрирует высокие показатели надежности (94%), чувствительности (93%) и специфичности (78%) и оценивает 4 типа приверженности: к лекарствам, изменению образа жизни, медицинскому сопровождению и лечению в целом [18]. В 2017 г. КОП-25 был признан и одобрен консенсусом Российского научного медицинского общества терапевтов [11]. Однако, несмотря на все преимущества, ряд авторов отмечает, что данный опросник является относительно затратным по времени заполнения, а также достаточно трудоемким для использования в условиях реальной клинической практики ввиду особенностей обработки результатов [16]. Кроме того, хотя данная методика в 2022 г. переведена на английский язык, опыта ее применения за рубежом пока нет, что ограничивает сопоставление данных научных исследований, проведенных в России с использованием КОП-25, с данными исследований других стран [18].

В настоящий момент существует более 40 валидизированных опросников и шкал, разносторонне оценивающих приверженность лечению. Однако следует признать, что до сих пор нет единой и признанной во всем мире методики, некоего «золотого стандарта», который был бы одобрен и рекомендован для проведения оценки приверженности широким кругом исследователей из разных стран и позволял бы систематизировать получаемые результаты [16][19]. Учитывая тот негативный вклад, который оказывает проблема недостаточной приверженности на результат лечения, особенно среди больных, страдающих хроническими заболеваниями, необходимы дальнейшие исследования с целью поиска и более широкого внедрения в клиническую практику оптимальной диагностической шкалы, отвечающей требованиям высокой надежности, чувствительности и специфичности.

Сахарный диабет — группа метаболических заболеваний, характеризующихся хронической гипергликемией и требующих пожизненного приема лекарственных препаратов. Помимо медикаментозной сахароснижающей терапии при СД2, большинству пациентов также необходим постоянный прием гипотензивных и гиполипидемических препаратов. Согласно современным клиническим рекомендациям, современная стратегия лечения СД1 и СД2 включает рациональное питание, физическую активность, самоконтроль гликемии и обучение принципам управления заболеванием.

Недостаточная приверженность пациентов лечению при СД может быть как следствием многофакторности самого заболевания, так и сложности выполнения целого комплекса терапевтических мероприятий, направленных на сохранение здоровья при данном хроническом заболевании. Основными барьерами приверженности при СД являются неэффективное взаимодействие врача и пациента, недостаточная мотивация пациентов в отношении изменения образа жизни и пожизненного приема большого числа лекарственных препаратов, а также недостаточная информированность пациента о своем заболевании вследствие неэффективной системы обучения. Другими причинами низкой приверженности лечению являются личностные и психологические особенности пациентов, клинические особенности течения заболевания, характер проводимого лечения, социально-экономические факторы [10]. Для улучшения приверженности среди пациентов с СД необходимо достижение определенного уровня взаимодействия пациента с медицинскими работниками. Правильное использование лекарственных препаратов и соблюдение рекомендаций по образу жизни требует от больных осознанного понимания важности и необходимости их регулярного приема, а также знаний о возможных рисках осложнений заболевания и возможностях их предотвращения [5][9].

## ЦЕЛЬ ИССЛЕДОВАНИЯ

Целью нашего исследования является проведение культурной адаптации «Опросника оценки приверженности медикаментозному лечению» 12-item Medication Adherence Scale (MAS-12) на русскоязычной выборке пациентов с СД.

## МАТЕРИАЛЫ И МЕТОДЫ

Данная диагностическая шкала была разработана Haruka Ueno в 2009 г. в объеме 14 вопросов и модифицирована автором до 12 вопросов в 2018 г. [19]. Опросник акцентирует внимание не только на соблюдении режима приема лекарственных препаратов, но и на психосоциальных факторах, включая оценку взаимопонимания между врачом и пациентом, понимания и принятия пациентом возможных побочных эффектов проводимой терапии и необходимости изменения образа жизни при СД. Опросник MAS-12 включает 4 субшкалы, состоящие из трех вопросов каждая: 1) соблюдение режима приема лекарственных препаратов, 2) сотрудничество с медицинскими работниками, 3) готовность получать и использовать информацию о лекарствах и 4) согласие принимать лекарства в соответствии с режимом и корректировать образ жизни. Каждая из шкал позволяет оценить соответствующий аспект приверженности лечению, а общий балл по 4 шкалам отражает уровень приверженности в целом. Каждый вопрос оценивается по 5-балльной шкале Лайкерта с вариантами ответов от 1 (никогда) до 5 (всегда). Баллы по пунктам каждой подшкалы суммируются для получения балла подшкалы, а общий балл приверженности лечению рассчитывается путем сложения всех 12 пунктов. По вопросам 3 и 12 баллы рассчитываются обратно. Более высокие баллы указывают на более высокую степень приверженности лечению. Первоначальный вариант опросника был признан в качестве показателя приверженности лечению при хронических заболеваниях (сердечно-сосудистых, сахарном диабете) в Японии. При проведении валидизации модифицированной версии опросника авторами в исследование были включены пациенты с хроническими заболеваниями, требующими длительного приема лекарственных препаратов (сахарный диабет 1 и 2 типов, ревматические заболевания, артериальная гипертония, дислипидемия и другие (болезни сердца, аллергии, включая астму, атопический дерматит и аллергические заболевания)). Внутренняя согласованность шкалы из 12 пунктов: коэффициент альфа Кронбаха составил 0,78 (0,74 для подшкалы «соблюдение режима приема лекарств», 0,81 для «сотрудничества с медицинскими работниками», 0,67 для «готовности получить доступ и использовать информацию о лекарствах» и 0,45 для «согласия принимать лекарства и того, как прием лекарств соответствует образу жизни пациента»). Шкалу могут использовать как пациенты самостоятельно для проверки соответствия выполнения данных им врачебных рекомендаций проводимому образу жизни и приему лекарственных препаратов, так и медицинские работники для лучшего понимания степени приверженности пациентов лечению. Помимо этого, эта шкала позволяет сравнивать приверженность лечению на разных этапах терапии с течением времени, а также облегчать оценку проведенной коррекции лечения. Предполагается, что данная относительно новая диагностическая шкала поможет людям с хроническими заболеваниями более эффективно контролировать свое лечение, а также улучшить качество жизни и долгосрочные результаты для здоровья. Возможно, в будущем эта шкала будет полезна не только в научных исследованиях, но и на практике в качестве удобного показателя оценки приверженности к лечению пациентов с различными хроническими заболеваниями.

## Место и время проведения исследования

Место проведения. Исследование проведено на базе клиники эндокринологии Университетской клинической больницы №2 Первого МГМУ им. И.М. Сеченова и с помощью интернет-ресурсов (GoogleForms) с сентября 2021 по август 2022 гг.

## Изучаемые популяции (одна или несколько)

Способ формирования выборки из изучаемой популяции (или нескольких выборок из нескольких изучаемых популяций).

Способ формирования выборки: простая случайная выборка.

## Дизайн исследования

одномоментное одновыборочное неконтролируемое исследование.

## Описание медицинского вмешательства (для интервенционных исследований)

Анкетирование. Среднее время заполнения опросника — 5–7 минут.

## Методы

Набор пациентов в исследование проводился эндокринологом на базе поликлинического отделения ЛДО №3 и эндокринологических отделений №1 и №2 УКБ №2 Первого МГМУ им. И.М. Сеченова. В исследование включались пациенты с СД1 и СД2, обратившиеся к эндокринологу или проходящие обследование и лечение в эндокринологическом стационаре, соответствующие критериям включения, подписавшие Информационный листок пациента.

Работа была выполнена в несколько этапов и включала процедуру культурной адаптации опросника, валидизации, проверку надежности и достоверности «Опросника оценки приверженности медикаментозному лечению», состоящего из 12 вопросов.

На первом этапе было получено разрешение на использование и проведение адаптации опросника у автора Haruka Ueno. Далее были выполнены перевод с английского языка и культурная адаптация выбранного опросника в соответствии с протоколом, разработанным ISPOR в 2005 г. [20]. Протокол включает подготовку, прямой перевод, создание объединенной версии, обратный перевод, проверку обратного перевода, согласование и коллективную оценку перевода, доработку, вычитку, заключительный отчет [21][22]. Вначале был выполнен прямой перевод исходной версии опросника с английского языка на русский. Перевод проводился тремя профессиональными переводчиками независимо друг от друга. Прямой перевод опросника включал вопросы к пациенту, варианты ответов, руководство по заполнению опросника для пациента и подсчету баллов для исследователя. Далее все версии перевода были проанализированы группой эндокринологов и переводчиков, на основании чего была создана объединенная версия. Затем был осуществлен обратный перевод объединенной версии с русского языка на английский профессиональным переводчиком с медицинским образованием. Группой врачей и переводчиков проведено сравнение оригинального опросника, первоначальных версий перевода, объединенной версии и обратного перевода. Внесены изменения. Окончательная версия была протестирована на группе из 10 пациентов с СД. Была проведена оценка понимания пациентами вопросов анкеты, сложностей при ее заполнении, после чего скорректированы формулировки вопросов и ответов с учетом замечаний респондентов. После коррекции обратный перевод был отправлен автору опросника.

Финальная версия опросника 12-item Medication Adherence Scale (опросник оценки приверженности медикаметозному лечению из 12 вопросов) (рис. 1).

**Figure:**
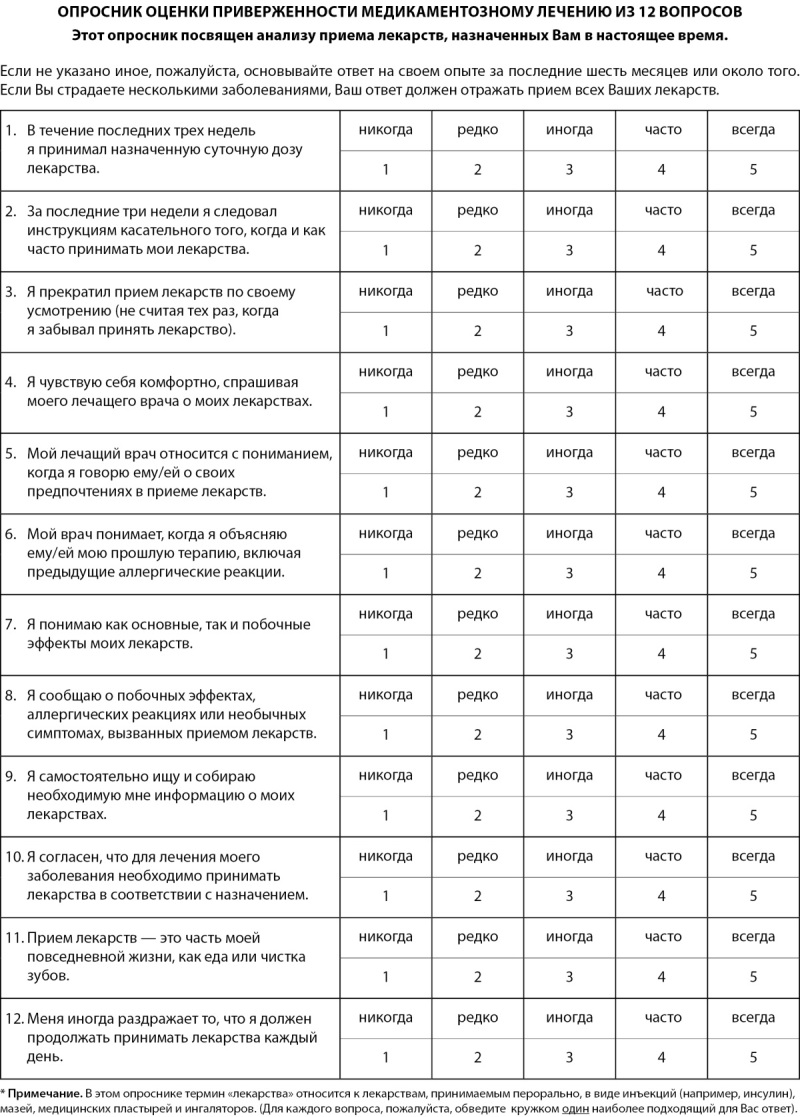
Рисунок 1. Опросник оценки приверженности медикаментозному лечению из 12 вопросов.

На 2 этапе проведена оценка воспроизводимости факторной структуры оригинального опросника на российской выборке и его психометрических свойств по следующим параметрам: валидность (конструктивная, конвергентная, текущая), внутренняя согласованность и ретестовая надежность.

Конвергентная валидность оценивалась при анализе корреляции шкал опросника MAS со шкалами опросника для количественной оценки приверженности к лечению (КОП-25) (Николаев, Скирденко, 2018).

## Статистический анализ

Статистический анализ данных проводился с использованием прикладной программы IBM SPSS Statistics, version 22 и IBM SPSS Amos version 20.

Для представления описательной статистики количественных показателей демографических и других характеристик включенных в исследование пациентов использовали среднее ± стандартное отклонение (СО) при незначительных отличиях от нормального закона распределения или медиану и квартили [Q1; Q3], если графические методы демонстрировали значительное отличие распределения значений показателя от нормального закона. Для описательной статистики по субшкалам опросника MAS-12 использовались параметрические и непараметрические статистические характеристики.

Качественные показатели представлены в виде абсолютных и относительных (долей в процентах) значений. Для характеристики тесноты линейной связи между показателями использовался непараметрический коэффициент корреляции Спирмена.

## Этическая экспертиза

Проведение исследования одобрено локальным этическим комитетом ПМГМУ им. И.М. Сеченова МЗ РФ (Сеченовский Университет). Исследование одобрено в рамках диссертационной работы. Выписка из протокола №08-19 от 05.06.2019.

## РЕЗУЛЬТАТЫ

Выборку составили 198 русскоязычных пациентов с диагнозом СД1 и СД2 в возрасте от 18 до 82 лет: 48 (24,2%) мужчин и 150 (75,8%) женщин. Среди них у 92 человек (46,5%) диагностирован СД1, у 105 человек (53%) — СД2 и у одного человека (0,5%) — панкреатогенный сахарный диабет. Длительность заболевания колеблется от нескольких месяцев до 45 лет и в среднем составляет 13,08 года.

Описательная статистика по субшкалам опросника ПМЛ-12, по данным включенных в обследование 198 пациентов, представлена в таблице 1.

**Table:** Таблица 1. Описательная статистика по субшкалам ПМЛ-12

	N	Среднее	Медиана	СО	Минимум	Максимум	Q1	Q3
Соблюдение режима приема лекарств	198	13,5	15,0	2,30	3	15	12,0	15,0
Сотрудничество с медицинскими работниками	198	12,3	13,0	3,21	3	15	10,0	15,0
Готовность получать и использовать информацию о лекарствах	198	11,8	12,0	2,35	3	15	11,0	10,0
Согласие принимать препараты в соответствии с режимом и корректировать образ жизни	198	12,8	13,0	1,96	6	15	11,75	15,0

## Оценка инвариантности факторной структуры опросника

Для проверки факторной структуры опросника оценки приверженности медикаментозному лечению 12-item Medication Adherence Scale использовалась процедура подтверждающего факторного анализа. Исходная модель, включающая все 12 утверждений, 4 фактора первого порядка и один фактор второго порядка, не продемонстрировала удовлетворительных показателей пригодности. После исключения из модели двух экзогенных переменных (пунктов опросника, для которых рассчитанные параметры структурной модели не давали статистически значимого вклада) показатели пригодности достигли требуемых значений (рис. 2 и табл. 2).

**Figure:**
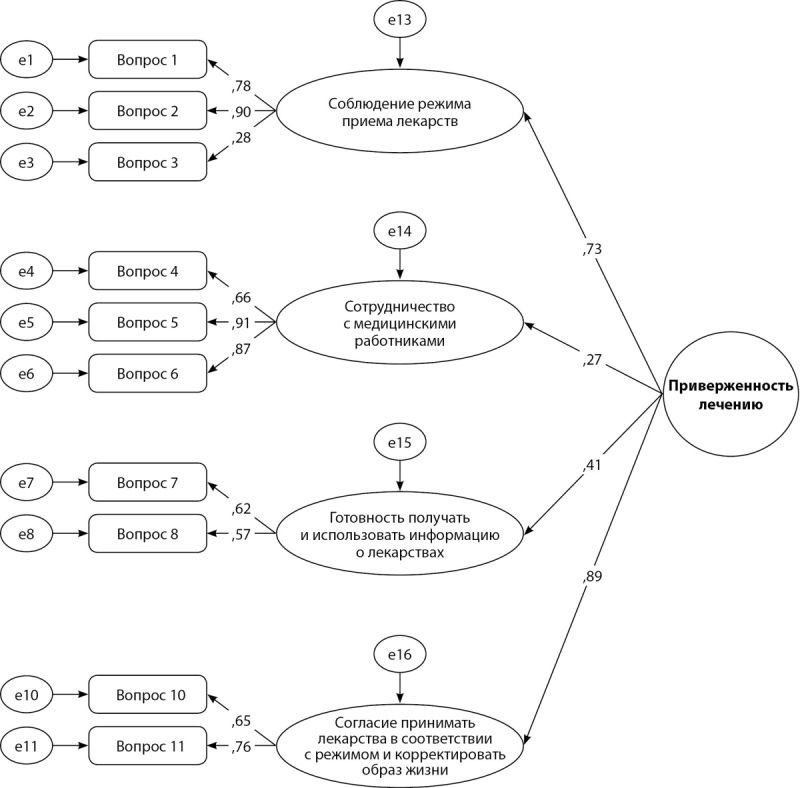
Рисунок 2. Факторная структура русскоязычной версии опросника ПМЛ при исключении вопросов 9 и 12. Примечание. На стрелках схемы указаны оцененные факторные веса.

**Table:** Таблица 2. Показатели пригодности исходной и итоговой моделей Примечание. CFI — Comparative Fit Index (сравнительный индекс соответствия); RMSEA — Root Mean Square Error of Aproximation (среднеквадратичная ошибка аппроксимации); AIC — Akaike Information Criterion (информационный критерий Акайке); TLI — Tucker-Lewis Index (индекс Такера-Льюиса).

	Хи-квадрат	p-value	CFI	RMSEA	AIC	TLI
Модель 1 (исходная)	120,888	0	0,898	0,089	180	0,856
Модель 2 (итоговая)	35,564	0,06	0,983	0,049	117,564	0,968

Оценка внутренней согласованности шкал опросника осуществлялась с помощью расчета коэффициента Альфа-Кронбаха. Результаты приведены в таблице 3.

**Table:** Таблица 3. Внутренняя согласованность шкал русскоязычной версии опросника «Опросник оценки приверженности медикаментозному лечению»

Шкала	Альфа Кронбаха
Соблюдение режима приема лекарств	0,659
Сотрудничество с медицинскими работниками	0,857
Готовность получать и использовать информацию о лекарствах	0,522
Согласие принимать препараты в соответствии с режимом и корректировать образ жизни	0,667
Приверженность лечению	0,766

## Оценка внешней валидности шкал опросника

Для оценки конвергентной валидности были рассчитаны коэффициенты корреляций шкал адаптируемого опросника со шкалами опросника КОП-25. Результаты приведены в таблице 4.

**Table:** Таблица 4. Взаимосвязи шкал опросников КОП-25 и ПМЛ-12, коэффициент корреляции (RhoСпирмена) Примечание: ** — статистическая значимость р<0,01, *— р<0,05.

КОП-25	Соблюдение режима приема лекарств	Сотрудничество с медицинскими работниками	Готовность получать и использовать информацию о лекарствах	Согласие принимать препараты в соответствии с режимом и корректировать образ жизни	Приверженность лечению
Важность	лекарственной терапии	0,198**	0,294**	0,240**	0,225**	0,333**
медицинского сопровождения	0,247**	0,245**	0,120	0,181*	0,294**
модификации образа жизни	0,094	0,225**	0,244**	0,062	0,228**
Готовность к	лекарственной терапии	0,327**	0,354**	0,268**	0,399**	0,423**
медицинскому сопровождению	0,313**	0,341**	0,155*	0,299**	0,357**
модификации образа жизни	0,290**	0,351**	0,140*	0,268**	0,353**
Приверженность	лекарственной терапии	0,302**	0,362**	0,294**	0,334**	0,426**
медицинскому сопровождению	0,319**	0,322**	0,143*	0,263**	0,362**
модификации образа жизни	0,212**	0,312**	0,228**	0,172*	0,322**
Приверженность лечению	0,304**	0,396**	0,263**	0,308**	0,431**

## Оценка ретестовой надежности шкал опросника

С целью оценки ретестовой надежности методики был проведен второй этап исследования, в котором принял участие 101 респондент из исходной выборки. В среднем между этапами проходило 2 месяца (интервал варьировался от 1 до 4 месяцев). Результаты приведены в таблице 5.

**Table:** Таблица 5. Оценка ретестовой надежности Опросника оценки приверженности медикаментозному лечению Примечание: ** — статистическая значимость р<0,01, * — р<0,05.

	Коэффициент корреляции (RhoСпирмена)
Соблюдение режима приема лекарств	0,567**
Сотрудничество с медицинскими работниками	0,630**
Готовность получать и использовать информацию о лекарствах	0,426**
Согласие принимать препараты в соответствии с режимом и корректировать образ жизни	0,535**
Приверженность лечению	0,611**

## ОБСУЖДЕНИЕ

Русскоязычная версия опросника оценки приверженности медикаментозному лечению — 12 (12-item Medical Adherence Scale) воспроизводит факторную структуру оригинального опросника, однако два вопроса из оригинальной шкалы не продемонстрировали статистически значимого вклада в третью и четвертую субшкалы опросника. Их исключение существенно улучшило показатели пригодности итоговой модели (CFI=0,983, RMSEA=0,049, TLI=0,968). Эти показатели свидетельствуют о хорошем соответствии эмпирических данных теоретической модели и позволяют сделать вывод об инвариантности факторной структуры оригинальной методики. Таким образом, на русскоязычной выборке рекомендуется использование опросника оценки приверженности медикаментозному лечению, состоящей из 10 вопросов (ПМЛ -10).

Анализ корреляций по 9-му вопросу («Я самостоятельно ищу и собираю необходимую мне информацию о моих лекарствах») показал наличие значимых отрицательных взаимосвязей с 4-м (r=-0,185, p=0,009) и 6-м (r=-0,219, p=0,002) пунктами, относящимися ко 2-й субшкале («Сотрудничество с медицинскими работниками»), что говорит о том, что во многих случаях самостоятельный поиск информации о лекарствах связан с недоверием лечащему врачу или нарушением коммуникации с ним. Соответственно, для российской выборки стремление и/или необходимость самостоятельно изучать информацию о принимаемых лекарствах могут быть связаны со снижением приверженности, в отличие от азиатской выборки, на которой апробировался оригинальный опросник. Возможно, чтобы нивелировать в российской популяции влияние фактора недоверия лечащему врачу при ответе на 9-й вопрос, его лучше переформулировать. Изучение результатов при скорректированной формулировке является задачей дальнейших исследований.

Для 12-го вопроса («Меня иногда раздражает то, что я должен продолжать принимать лекарства каждый день») были выявлены слабые, хотя и статистически значимые, положительные коэффициенты корреляции с 4-м (r=0,213, p=0,003) и 5-м (r=0,192, p=0,007) пунктами опросника, а также отрицательная корреляция (r=-0,234, p=0,001) с 9-м пунктом. То есть повышение приверженности в части сотрудничества с медицинским персоналом связано с несколько большей частотой негативных переживаний по поводу необходимости приема лекарственных препаратов, а активный самостоятельный поиск информации о лекарствах, напротив, ассоциируется со снижением негативных переживаний по поводу необходимости их приема. Здесь, по нашему мнению, опять же наблюдается характерное для российской выборки — в сравнении японскими пациентами — недоверие к медицинскому персоналу и системе здравоохранения в целом.

Внутренняя согласованность шкал адаптируемой методики, оцененная с помощью коэффициента альфа Кронбаха, продемонстрировала достаточный уровень внутренней согласованности по шкале в целом (0,766) и разный уровень надежности для отдельных субшкал (от 0,522 для субшкалы «Готовность получать и использовать информацию о лекарствах» до 0,857 для субшкалы «Сотрудничество с медицинскими работниками»).

Оценка конвергентной валидности с помощью корреляционного анализа между шкалами адаптируемой методики и опросника КОП-25 продемонстрировала значимые корреляции между большинством субшкал данных методик. Наиболее тесно связаны оказались субшкалы «Готовность к лекарственной терапии» и «Приверженность к лекарственной терапии» опросника КОП-25, а также общий показатель по этой методике с общим показателем приверженности лечению по адаптируемой методике.

Оценка ретестовой надежности продемонстрировала значимые корреляции по всем субшкалам опросника и по общему баллу, находящиеся в диапазоне от 0,426 до 0,630 (p<0,01). Такие показатели обычно не рассматриваются как достаточные, однако стоит учесть тот факт, что приверженность лечению не выступает как устойчивое личностное свойство и может меняться с течением времени под влиянием различных факторов.

Данные проведенного нами исследования были обсуждены с автором опросника 12-item Medical Adherence Scale Haruka Ueno. Получено одобрение автора и его коллег на модификацию оригинального опросника до 10 вопросов с условием использования данной шкалы только на территории России. Итоговая шкала, таким образом, включает 10 вопросов (рис. 3).

**Figure:**
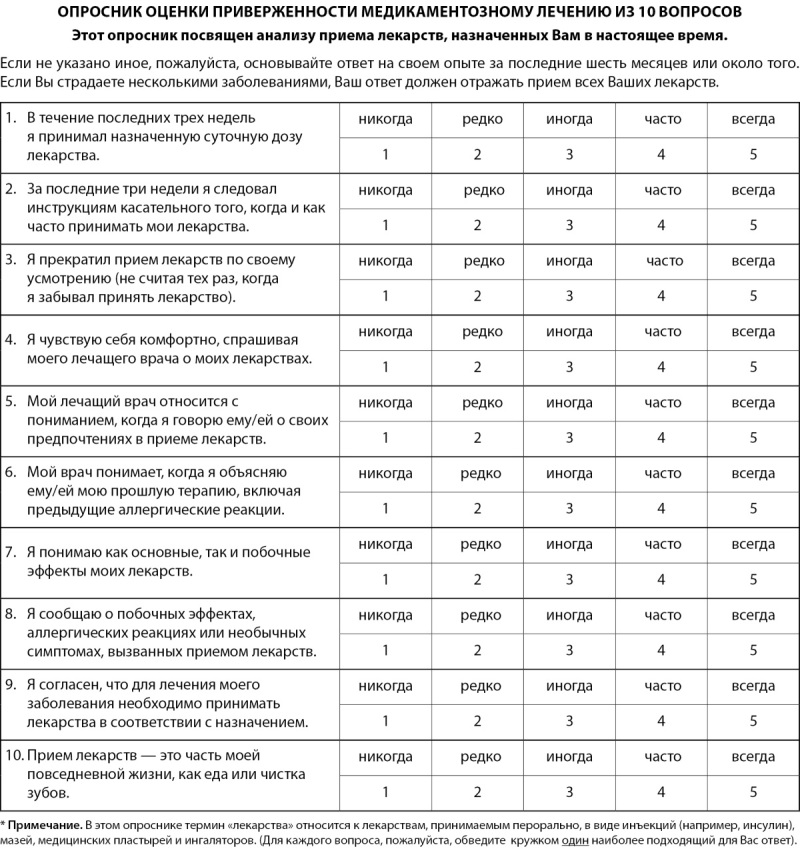
Рисунок 3. Опросник оценки приверженности медикаментозному лечению из 10 вопросов.

## Репрезентативность выборок

Выборка исследования не была однородной с точки зрения условий проведения тестирования: часть пациентов обследовалась в стационаре, где некоторые из них проходили мотивационное интервью с целью повышения приверженности лечению, в то время как другая часть пациентов наблюдалась амбулаторно. Таким образом, у части выборки могло наблюдаться изменение уровня приверженности лечению, в то время как для другой ее части показатели первого замера могли остаться неизменными, что в свою очередь отразилось на величине рассчитанных коэффициентов корреляции.

## Сопоставление с другими публикациями

Русскоязычная версия Опросника оценки приверженности медикаментозному лечению-12 (12-item Medical Adherence Scale) не публиковалась ранее в нашей стране. Данная шкала была разработана Haruka Ueno в объеме 14 вопросов и модифицирована автором до 12 вопросов в 2018 г. Опросник и в настоящее время используется в Японии как для оценки приверженности при сахарном диабете, сердечно-сосудистых заболеваниях, так и при валидизации зарубежных опросников, а также в других странах, включая Саудовскую Аравию и Китай [23][24].

## Ограничения исследования

Говоря об ограничениях настоящего исследования, в первую очередь стоит отметить однородность выборки в отношении основного заболевания (пациенты с СД). Использование данной методики для оценки уровня приверженности у пациентов с другими хроническими заболеваниями требует дополнительного изучения на соответствующих группах. Также, для более объективной оценки чувствительности методики в качестве внешнего критерия, помимо методов самоотчета, необходимо сопоставить результаты, полученные в ходе анализа опросника, с данными объективного наблюдения, что, конечно, связано с рядом серьезных затруднений, основное из которых — осведомленность участников таких исследований о факте наблюдения. Кроме прочих ограничений исследования, следует отметить, что обратный перевод проводился не носителем языка, а русскоязычным переводчиком.

## Направления дальнейших исследований

Русскоязычную версию опросника оценки приверженности медикаментозному лечению (ПМЛ 10) (12-item Medical Adherence Scale) в дальнейшем планируется использовать для изучения приверженности лечению на более широкой выборке пациентов с сахарным диабетом, а также изучить возможность ее применения среди пациентов, страдающих другими хроническими заболеваниями (гипертоническая болезнь, сердечно-сосудистые заболевания).

## ЗАКЛЮЧЕНИЕ

Факторная структура адаптируемого оригинального опросника 12-item MAS впервые воспроизводится на российской выборке пациентов с СД1 и СД2 и демонстрирует хорошие показатели пригодности при исключении двух вопросов из 3-й и 4-й субшкал, что указывает, по нашему мнению, на культурные особенности российской выборки, а именно более низкий уровень доверия медицинским работникам и системе здравоохранения в целом. Полученные корреляции со шкалами российского опросника КОП-25 подтверждают внешнюю валидность адаптируемой методики, следовательно, возможность ее использования в научной и практической работе с пациентами с СД. Модификация опросника 12- item MAS до 10 вопросов согласована с автором Haruka Ueno при условии его использования в Российской Федерации. Данный опросник оценки приверженности медикаментозному лечению» (ПМЛ-10), состоящий из 10 пунктов, обладает хорошими психометрическими свойствами, является валидным и надежным инструментом для оценки приверженности лечению среди больных с СД1 и СД2 и может быть рекомендован к применению в клинической практике.

## ДОПОЛНИТЕЛЬНАЯ ИНФОРМАЦИЯ

Источники финансирования. Исследование выполнено в рамках проведения научно-исследовательской работы. Работа выполнена по инициативе авторов без привлечения финансирования.

Конфликт интересов. Авторы декларируют отсутствие явных и потенциальных конфликтов интересов, связанных с содержанием настоящей статьи.

Участие авторов. Епишин В.Е., 1 — вклад автора 1 по критерию 1 — в концепцию и дизайн исследования, в получение, анализ данных и интерпретацию результатов, по критерию 2 — написание статьи; Калашникова М.Ф., 2 — вклад автора 2 по критерию 1 в концепцию и дизайн исследования, получение данных, интерпретацию результатов по критерию 2 — написание статьи; Лиходей Н.В., 3 — вклад автора 3 по критерию 1 в концепцию и дизайн исследования, получение данных, интерпретацию результатов, по критерию 2 — написание статьи; Бондарева И.Б., 4 — вклад автора 4 по критерию 1 — в анализ данных и интерпретацию результатов, по критерию 2 — написание статьи; Каурова А.М., 5 — вклад автора 5 по критерию 1 — в получение, анализ данных, интерпретацию результатов, по критерию 2 — внесение в рукопись существенной (важной) правки с целью повышения научной ценности статьи; Тулупова М.В., 6 — вклад автора 6 по критерию 1 — в получение, анализ данных, интерпретацию результатов, по критерию 2 — внесение в рукопись существенной (важной) правки с целью повышения научной ценности статьи; Николаев Н.А., 7 — вклад автора 7 по критерию 1 — в концепцию исследования, по критерию 2 — внесение в рукопись важной правки с целью повышения научной ценности статьи; Фадеев В.В., 8 — вклад автора 8 по критерию 1 — в концепцию исследования, по критерию 2 — внесение в рукопись важной правки с целью повышения научной ценности статьи.

Все авторы одобрили финальную версию статьи перед публикацией, выразили согласие нести ответственность за все аспекты работы, подразумевающую надлежащее изучение и решение вопросов, связанных с точностью или добросовестностью любой части работы.

Благодарности. Авторы выражают благодарность автору опросника MAS 12, доктору Haruka Ueno, MPH, PhD, Department of Health and dietetics, Faculty of Health and Medical Science, Teikyo Heisei University за разрешение использовать и модифицировать опросник, а также Ю.П. Сыч, к.м.н., асс. кафедры эндокринологии лечебного факультета №1 за техническую помощь и перевод текста.
